# On an Eruptive Disease of Children

**Published:** 1808-04

**Authors:** Whitley Stokes

**Affiliations:** Professor of the Practice of Medicine


*On an eruptive Disease of Children. By* WHITLEY STOKES, M. D. S. F. T. C. D. *Professor of the Practice of Medicine.*

[ Extracted from the Dublin Medical Essays. ]

A Very severe disease is to be met with occasionally, among children, in various parts of Ireland; and, if I can depend on the information of unprofessional persons, in England also. This is an eruption of vesicles often behind the ears, followed by ulcers with copious discharge, loss of substance, and rapid tendency to mortification.

If a description of this disease has been published, I have not happened to see it; and although I do not pretend to have ascertained its history precisely, yet I hope I can give such an outline as may enable practitioners to combine their observations on a disease, which, though not very frequent, is so much so, considering its great severity, as to require a degree of attention it does not seem to have hitherto received.

This disease is known by various denominations in different parts of Ireland' as might be expected. In the counties of Dublin and Wicklow it is called, the white blisters; in in the counties of Down, Antrim, Derry, and Monaghan' the eating hive; in the counties of Waterford and Tipperary, the burnt holes: I would propose to call it Pemphigus gangrænosus.

The approach of this disorder is sometimes, though rarely, denoted by a livid suffusion like that of erysipelas slightly elevated. This was both observed by Dr. M'Donnel of Belfast, and by Dr. Spear, in the county of Monaghan, at a time when the disease prevailed there epidemically. It more frequently happens, however, that the complaint comes on in perfect health. One or more vesicles appear mostly larger than the best distinct small-pox; these increase for two or three days, burst, and discharge a thin fluid having a disagreeable smell, limpid in most cases, sometimes whitish and sometimes yellowish, the latter less dangerous; usually the weaker the child's constitution is, the thinner is the matter. Before or after breaking, the vesicles run together, the sore becomes painful, with loss of substance and a thin fœtid ichorous discharge, the edges of the ulcer are undermined, and it spreads quickly.

The more usual seats of the disease are, behind the ears, sometimes on the hands or feet, on the private parts, (seldom on the arm-pit,) the breast, folds of the thighs, lower belly, on the inside of the mouth or lips. The disease, however, it is said, seldom passes from the inside to the outside of the mouth.

In the progress of the disorder, the ulcers enlarge rapidly, with remarkable fœtor, very great discharge, and livid edges.

If the sores are behind the ears, they destroy the connection of the posterior cartilage with the cranium; they spread to the meatus auditorius; to the eyes, the sight of which seemed, in a few cases, to have been destroyed one or two days before death; and they sometimes extend to the vertex.

The constitutional disturbance, that accompanies this disease, seems principally the effect of irritation. When the vesicles burst, the child begins to grow peevish and fretful, pale, loses its appetite, and the flesh becomes remarkably flabby. The periods of the disorder are not very regular; but it often happens, about the eighth day, that the pulse sinks, the lividity spreads over the whole sore, the fœtor and discharge increase greatly. The smell is so strong, as often to be perceivable at a distance from the bed. The discharge, in one case, where the ulcers affected fected the arm-pits and breasts, was such, that the linen was completely loaded several times a day.

Death takes place about the tenth or twelfth day, often preceded by convulsions, sometimes by extreme debility. Patients are apt to relapse soon after the sores are skinned over.

The causes of this malady are rather obscure. It seems exclusively confined to children. Dr. M'Donnel saw twenty cases before the year 1795; all the patients were under four years old. Dr. Spear observed, that it was confined to children from the age of three months to that of five years; but it has been observed, near Dublin, in children of nine years old. It attacks the finest children in preference; the children of the poor more frequently than those of the affluent; and those, who live in damp situations, seem more peculiarly subject to it than others. The disease is more prevalent in summer than in winter. It appears to be infectious, though obscurely so, in general; but, in the year 1800, Dr. Spear observed it to spread epidemically. It has been said, that the disease oftener affects the younger from the older than the reverse. It would be interesting to determine, whether it attacks the same person twice; it certainly is apt to return after apparent recovery. Children, as is well known, are very subject to excoriations behind the ears, which sometimes produce formidable sores; these may, possibly, in a few cases, resemble the disease we speak of in its advanced stages; but, in a great majority of cases, these excoriations are far less rapid and dangerous than the complaint in question.

On the other hand, the swine-pock (varicella) resembles this disease in its first stage; but the fever rarely precedes the eruption in white blisters, and pustules of varicella dry readily.

This is a disorder of great danger, but of various progress in different individuals. It often happens, that a fatal change takes place about the eleventh day. The unfavourable signs are, the rapidity with which the sores spread; the blackness, first at the edges, after some time spreading over the whole; the quantity and fœtor of the discharge; its colour, the paler being the most dangerous.

It has been alledged, by empirical practitioners in this disease, that, after the blackness had covered the whole sore, death was certain; but I have observed the blackness to go off, although it had spread over the whole surface of the sores. When this appearance abates, livid streaks generally remain for a day or two. When a favourable change change is effected, in bad cases, the diminution of the fœtor and discharge were the first signs of the abatement of the malady; appetite was afterwards restored.

My first information, with respect to this disease, came from Dr. James M'Donnel, of Belfast, whose zeal for procuring original information in medicine and natural history is well known. His observations were first mentioned to me by the late Dr. Thos. Cleghorn, in 1792, in Edinburgh. On my return to Ireland, I had an opportunity of making many enquiries from Dr. M'Donnel, respecting this complaint, and obtained, with other interesting particulars, the following:—that it was not described in any book he had consulted; that it was very fatal; little understood by physicians or surgeons in that part of Ireland; and that there were solid grounds for supposing, that a traditional treatment, of which the use of some green vegetable substance, made into an ointment, was the principal part, was effectual in stages of the disease not very far advanced.

Some years afterwards I learned from Dr. Barker, that a similar disease prevailed in the county of Waterford; and that it was also the prevailing opinion there, that it was more successfully treated by women, who were acquainted with some traditional methods, than by regular practitioners. Soon after my commencement in practice, two cases of this disease, distinctly marked, came under my care; they both proved rapidly fatal. The second of these cases was peculiarly distressing; it was the only child of a near relation. I warned the parents of the nature of the complaint, and had the assistance of an eminent practitioner. Both these children were treated by the external application of bark.

Excited by those events, I now enquired more assiduously after the traditional methods. I discovered a woman, of the name of Murray, who was reported to have succeeded in several severe cases. I prevailed on her to let me see some of the cases in progress; and being satisfied that she succeeded well, I procured her method, and took notes of such symptoms as she had observed. I also obtained several recipes for the green ointment, from different parts of the county of Wicklow, most of which agreed more or less with Murray's.

Some cases soon occurred to myself, and for a considerable time my success was uniform. The ointment I used was prepared by Murray. I mentioned, each year, the result of my enquiries to the gentlemen who attended my Lectures on the Practice of Medicine. In 1802, Mr. Quin, who who then attended, mentioned his having seen the disease often, under the care of Dr. Spear, of Glasslough, in the county of Monaghan. At my request, Mr. Quin wrote to Dr. Spear, who favoured me with a very full description of the disease; and confirmed, by the experience of his neighbourhood, the accounts, which had been received from other parts of Ireland, of the frequent success of the traditional treatment.

I was tempted to depart from my instructors, so far as to substitute a simple composition for the very complex one I had been taught the use of. Murray's ointment, by her account, was thus made; equal quantities of the following plants were taken. I have taken some pains to ascertain the plants she meant, by making her minutely describe them, and, when necessary, by examining the plant she used; so that I hope there is little error in that respect. They were,

| | |
|---|---|
| Ground ivy . . . . . . | Glecoma hederacea. |
| John's-wort . . . . . . | Hypericum androsæmum. |
| Rue . . . . . . . . . . . | Ruta graveolens. |
| Holyhock . . . . . . . | Alcea rosea |
| Bark of elder . . . . . | Sambucus nigra. |
| Rib grass . . . . . . . . | Plantago lanceolata. |
| Broad plantain . . . . | Plantago major. |
| Phoghram . . . . . . | Scrophularia nodosa. |
| Penny leaf . . . . . . | Cotyledon umbilicus. |
| Woodbine . . . . . | Lonicera periclymenum. |
| Elecampane . . . . . | Inula helenium. |
| Tansy white . . . . . | Potentilla anserina |

As I had also many other recipes for making a green vegetable ointment, and had good grounds to suppose, that several different compositions of this kind were used with success, I determined to make an ointment of a single vegetable; and, in selecting that vegetable, I was directed by its occurring in many recipes of this kind, and having itself a character among the common people, as a useful application to obstinate ulcers. Upon this ground, I fixed on the scrophularia nodosa, called, in the north of Ireland, rose noble; in the south, phoghram, pronounced phoram; in England, great fig-wort. This simple ointment, I hope, is as powerful as the compound, although my success with it has not been uniform; but the failures, which have been very few in comparison of the successful cases, have been those not under my own eye, or very far advanced: and I have reason to know, that no person is so successful as to be sure of rescuing the patient in all stages.

The

The following method is nearly what I have pursued for several years past, and what I advise. When the parts adjoining the sores are swelled, and strongly suffused with a dusky redness, or if the sores have been previously dressed by any dry powder, I apply a poultice of porter and oat-meal. The carrot poultice, in fermentation, if it can be procured without any delay, would perhaps be useful. After about eight hours, the poultice should be removed, and the parts affected very gently wiped with a roll of lint or soft rag; then the scrophularia ointment should be applied. It should be as highly saturated with green vegetable matter as possible. For this purpose, the plant should be taken fresh, the smaller leaves selected, and stewed a considerable time with as small a quantity of unsalted butter as will be sufficient to prevent the leaves from being scorched. If well prepared, it is of a full grass green colour; but after keeping, it becomes the colour of box leaves, especially at the surface; yet I apprehend it preserves its efficacy, in a great degree, for many months. When applied it should be melted, and suffered to cool to the consistence of honey; it should be applied by a soft feather, with the utmost gentleness, to the whole surface of the sore. Through the whole of the treatment, the greatest gentleness should be used. If the ear is strongly drawn open, the parts affected are made to bleed, which produce many inconveniencies, and retards the progress of the cure. After smearing the ulcer with the ointment, it should be dressed with the same ointment, with the addition of one-eighth part of wax. This last ointment should be spread on lint folded to the dry side, and cut so as to fit behind the ear; the whole should be secured by a broad bandage, drawn under the chin, and fastened over the top of the head. This dressing, in very severe cases, should be repeated every fourth or sixth hour; but, as the fœtor abates, larger intervals may be allowed. Hairs should be completely removed from the neighbourhood of the sore. I believe it to be necessary, that the child's bowels should be kept open. I also direct the internal use of yeast, which I am of opinion is of service, but cannot decidedly prove it to be so.

Such is the treatment I propose for this disease, selected from such parts of the traditional method of cure as seem to have been most successful, with a very little addition. I do not pretend that this treatment will always succeed; but perhaps of four such cases as may occur to any practiotiner, not excluding the most hopeless, it will succeed in three three on an average: and if we could accurately ascertain the mortality which takes place when the other methods are used, we should consider this proportion of success as very satisfactory. I have had a variety of opportunities of concluding, with a high probability, that the treatment I recommend is superior to any of the following applications. The carrot poultice, although this, I doubt not, has been of use in a few cases; preparations of mercury, of lead, of zinc; powder of bark, of starch; washing with brine, with soap and water. I have named these in the order in which they are more and more objectionable. The first I believe to have been useful; although I have pretty well ascertained, not so useful as the green vegetable ointment; the last I have some reason to believe to have been pernicious.

The utility of the scrophularia ointment does not stand on my testimony alone. I have annually mentioned, at Lecture, the state of my observations on this disease, and I have received favourable accounts of the use of it from several friends; some engaged in the profession, and some not. Mr. Creighton, surgeon to the Foundling Hospital, particularly authorises me to state, that he used it for upwards of a year with uniform advantage.

I do not pretend to have proved, that the scrophularia has a specific effect on this disease; possibly it is only useful, by supplying the green vegetable matter; but as I and some of my friends have had a success more uniform, by means of this ointment, in a disease in which our failure was almost uniform before, I shall continue to prefer it to other vegetable matters until the subject is better understood.

*Trinity College, Dublin, June* 15, 1807.

